# Nephroprotective Effects of *Alhagi camelorum* against Cisplatin-Induced Nephrotoxicity in Albino Wistar Rats

**DOI:** 10.3390/molecules27030941

**Published:** 2022-01-29

**Authors:** Muhammad Omer Iqbal, Muhammad Masood Ahmed, Shafia Arshad, Usman Javaid, Imran Ahmad Khan, Majid Manzoor, Shumaila Andleeb, Romana Riaz, Shaukat Hussain Munawar, Zahid Manzoor, Asma Mumtaz

**Affiliations:** 1 Shandong Provincial Key Laboratory of Glycoscience and Glycoengineering, School of Medicine and Pharmacy, Ocean University of China, Qingdao 266003, China; 2 Fatima Tu Zahara Department of Life Sciences, Muhammad Institute of Medical and Allied Sciences, Multan 60000, Pakistan; 3 College of Pharmaceutical Sciences, Zhejiang University, Hangzhou 310058, China; masoodkarni@yahoo.com (M.M.A.); dr.majidmanzoor@yahoo.com (M.M.); 4 Faculty of Pharmaceutical Sciences, Times Institute Multan, Multan 60000, Pakistan; 5 Faculty of Medicine and Allied Health Sciences, The Islamia University of Bahawalpur, Bahawalpur 93100, Pakistan; shafia.arshad@iub.edu.pk; 6 Department of Pharmacology, Faculty of Pharmacy, Bahauddin Zakariya University, Multan 60800, Pakistan; usmanjavaidkhan1@gmail.com; 7 Department of Pharmacology, The Islamia University of Bahawalpur, Bahawalpur 63100, Pakistan; 8 Southern Punjab Institute of Health Sciences, Multan 60800, Pakistan; drshama2010@live.com; 9 Department of Pharmaceutics, Faculty of Pharmacy, Bahauddin Zakariya University, Multan 60800, Pakistan; rummanariaz@yahoo.com (R.R.); asmahashmi83@gmail.com (A.M.); 10 Department of Pharmacology and Toxicology, Cholistan University of Veterinary and Animal Sciences, Bahawalpur 63100, Pakistan; zahidmanzoor@cuvas.edu.pk; 11 Multan Medical and Dental College, Multan 60000, Pakistan

**Keywords:** cisplatin, nephrotoxicity, reactive oxygen species, *Alhagi camelorum*

## Abstract

*Alhagi camelorum* (AC) is an old plant with a significant therapeutic value throughout Africa, Asia, and Latin America. The overuse of cisplatin (Cis > 50 mg/m^2^) is associated with observed nephrotoxicity, ototoxicity, gastrotoxicity, myelosuppression, and allergic reactions. Remedial measures are needed for the protection of nephrotoxicity against cisplatin. Thus, we investigated the nephroprotective effects of AC plant extract to prevent cisplatin-induced nephrotoxicity in albino Wistar rats. The presence of polyphenols, phenolic compounds, tannins, and saponins was revealed during phytochemical investigation, and a significantly intense antioxidant activity was recorded. There were no toxicological symptoms in the treated rats, and no anatomical, physiological, or histological abnormalities were found compared to the control rats. The results of correcting cisplatin-induced nephrotoxicity revealed that the extract has a significant ability to treat kidney damage, with most parameters returning to normal after only three weeks of therapy. It is concluded that co-administration of cisplatin with AC extract showed exceptional nephroprotective effects at a dose of 600 mg/kg for Cis-induced nephrotoxicity.

## 1. Introduction

According to emerging evidence, nephrotoxicity is one of the most persistent kidney problems with an 8–15% lifetime risk in Europe, 2–5% in Asia, and 20% in the Middle East [1]. Nephrotoxicity leads to a reduction in the glomerular filtration rate and an increase in creatinine and blood urea nitrogen in the serum, ultimately increasing the blood pressure and fluid retention in the body (over-hydration) [1,2]. Kidneys are the primary target organ to bear toxic effects of medication. Kidneys account for 25% of the heat output and are naturally exposed to circulatory drugs and chemicals as central excretion bodies. These nephrotoxic drugs contribute to acute kidney failure and increased morbidity and death [3,4]. Because of their functions in glomerular concentrations, drug delivery, and metabolism, the epithelial cells of the renal proximal convoluted tubules (PCT) are a crucial target for nephrotoxicants [5]. Nephrotoxic agents usually damage the renal tubular epithelial cells either by reacting indirectly (through metabolites) or directly with membrane components and cellular macromolecules [6,7]. 

Cisplatin (Cis) is the most commonly used potential chemotherapeutic agent against different solid tumors, including those in the head, neck, lung, breast, bladder, and ovary. Besides its multiple advantages, Cis is responsible for inducing several side effects, including ototoxicity, gastrotoxicity, myelosuppression, and allergic reactions [8,9]. According to emerging evidence, the main toxic effect of Cis is the dose-limiting nephrotoxicity that is responsible for mortality and morbidity [10,11]. Several previous studies indicated the nephrotoxicity of Cis at a single dose (50–100 mg/m^2^) [9,12]. Nephrotoxicity caused by Cis occurs mainly in renal PCT [13].

Nephrotoxicity is the most common adverse effect of Cis accumulation in kidneys after chemotherapy [14]. The Cis disturbs the equilibrium between antioxidants and peroxides, while renal fibrosis is closely related to a rise in oxidative damage [15,16]. The Cis-complex moves through the cell membranes in a unionized form due to its high chloride concentration in the plasma. Cl-plasma is higher than the intracellular concentration, and chloride ligands are displaced by water, resulting in a nephrotoxic formation of the positive platinum complexes. The Cis molecule binds to the guanine DNA base and inhibits DNA, RNA, and protein synthesis. Cis binds to the DNA interface, and an intrastrand is established, leading to a faulty genetic code model and the arrest of the formation and duplication of DNA replication [17,18].

During the past few decades, natural compounds have been considered among the promising therapeutic agents against cancer, cardiovascular diseases, aging, diabetes, and especially neurodegenerative disorders due to their wide variety of modes of action, efficiency, accuracy, and fewer side effects [19,20]. Several studies have focused currently on traditional herbal medicines to evaluate novel therapeutic drugs for acute kidney injury (AKI) therapy. Various herbal medicines, including pomegranate (Lythraceae), *Prosthechea michoacana* (Orchidaceae), *Zingiber officinale* (Zingiberaceae), and red ginseng (family Araliaceae), have protective effects against cisplatin-induced acute kidney injury with vivo experiments [21,22,23].

*Alhagi camelorum* (AC) is a traditional herb that belongs to the family Leguminosae [24]. AC is used to treat metabolic, digestive, and hepatic problems, autoimmune diseases, headaches, and infections [25]. AC treats stomach problems of animals, heartworm, and pyrexia [21,22,23]. The herb is regarded as a laxative, diuretic, purgative, and antipyretic [26]. The major phytonutrients in AC include proteins, glycosides, coumarins, flavonoids, phenolics, resin, saponins, steroids, terpenes, ascorbic acid, essential oils, salicylic acid, ascorbic acid, and gallic acid [27]. This drove us to explore the nephroprotective effect of AC against chemotherapeutics such as cisplatin.

However, to the best of our knowledge, the in vivo toxicological effect and the nephroprotective effects of AC plant extract have not been identified. The current nephroprotective study serves as a necessary basis for further studies developing herbal medicine from this plant.

## 2. Materials and Methods

### 2.1. Chemicals

The analytical-grade chemicals included cisplatin (Mylan S.A.S, CHATILLON SUR CHALARONNE, AUVERGNE RHONE ALPES, France), 2, 2-Diphenyl, 1-picrylhydrazle, formalin, ketamine, and xylazine, which were purchased from Prix Lab Lahore, Pakistan. Ethanol (99.2% pure), picric acid (99.5% pure), NaOH, and trichloroacetic acid (TCA; 97% pure) were obtained from Sigma-Aldrich, St. Louis, MO, USA. The chemicals were mixed with other chemicals and with distilled water depended on the parameters and their protocols.

### 2.2. Preparation of the Plant Extract

AC was collected from the agricultural fields of Head Muhammad Wala, Multan, Pakistan. The plant was authenticated by expert taxonomists in the Department of Botany, Bahauddin Zakariya University, Multan, Pakistan, with the voucher number (R.R. Stewart F.W. Pak.711/12) for further reference. AC plants were washed, dried (under a shield), and powdered with the help of a herbal blender. AC powder (1200 g) was soaked in a hydroalcoholic solvent (70:30 *v*/*v*) in an air-tight amber-colored bottle for nine days. A rotary evaporator (Heidolph Laborota 4000 efficient, Hamburg, Germany) was used to evaporate the filtrate at reduced pressure [28,29]. The obtained semi-solid residue was refrigerated before further analysis.

### 2.3. Animals

Albino Wistar male rats weighing 260–290 g were collected from the animal house of the Department of Life Sciences, Muhammad Institute of Medical and Allied Sciences, Multan, and kept in polycarbonate cages that were covered by raw dust that was changed every three days under standard laboratory conditions (27 ± 2 °C) in the Pharmacology Research Laboratory. The rats were given water and standard diet pellets *ad libitum*. All experiments performed were approved by the Animal Ethical Committee of the Muhammad Institute of Medical and Allied science, Multan, Pakistan [30], in accordance with the guidelines of the national research council [31].

### 2.4. In Vivo Experiments

A single dose of Cis (5 mg/kg) was injected into the rats to induce nephrotoxicity [32]. Rats were typically divided into four different groups of six animals each. Group-I was given normal saline by oral gavage for 21 days and used as a control. Group-II received Cis 5 mg/kg (i.p) on the first day and received saline for 21 days by oral gavage. Group-III was given Cis + AC extract (400 mg/kg; cisplatin was given on the first day and then the extract was given for 21 days by oral gavage), while Group-IV received Cis + AC extract (600 mg/kg; cisplatin was given on the first day and then the extract was given for 21 days by oral gavage). The plant extract was freshly suspended in distilled water before administration with the aid of Tween 80. The rational choice of dose and treatment time was based on a previous study on different plants [30].

Daily food and water consumption was regularly measured with the body weights of the rats. The body weight was measured before the experiment, while kidney weight was measured by sacrificing the rats. The urine was collected on days 0, 7th, 14th, and 21st from all experimental groups of rats by placing each rat on a plastic dish for sodium (Na), potassium (K), and creatinine level analysis [33]. The rats were kept for 24 h in metabolic cages with tap water, and we measured the total intake of water and the amount of urine. The collected urine samples were stored at −30 °C for the estimation of creatinine, Na, and K levels. Similarly, the blood samples of all experimental groups were collected on days 0, 7th, 14th, and 21st in EDTA tubes and centrifuged at 2300 rpm to collect the plasma to estimate the creatinine, Na, and K levels. 

### 2.5. Phytochemical Screening

Phytochemical screening of the secondary metabolites and active compounds present in the AC extract was done using the standard protocols [34].

### 2.6. HPLC Analysis

The standard USP and ICH guidelines were carried out for the HPLC analysis to estimate phenolic acids and polyphenolic compounds [35]. The wavelength used for polyphenol identification was 280 nm, while the temperature of the furnace column was adjusted to 35 °C. Ultimate 3000 liquid chromatography incorporating a 5 cm flow cell DAD and Chromeleon system management for HPLC analytics were used. The reversed-phase Acclaim C18 column (5-micron particle size, 250 mm/4.6 mm) was used to differentiate components. In total, 30 mg dry methanol and water extract were dissolved separately in 25 mm of the mobile phase solvent. The 0.45 μm membrane filter filtered the sample solution before injection (methanol: 0.5% acetate acid in water: 1.9) in the HPLC system. High-performance liquid chromatography analysis was conducted using the methanol phase containing the mobile solvent (Solvent A) and acetic acid solutions (Solvent B), with 105 min for each sample. The HPLC spectrum library recorded and stored each standard. With respect to the identification criteria for *Alhagi camelorum*, chemicals were determined when the retention time and spectrum of unknown compounds were compared to the HPLC standard library. In the extracts, phenolic acids and flavonoids were measured by applying the calibration graph by drawing spikes against the relevant standard control sample. The data are reported as the standard ± error means for three independent assessments.

### 2.7. Acute Oral Toxicity Dose Test

The acute oral toxicity of AC was evaluated in 12 rats. Rats were divided into six groups; each group contained four rats. Rats were fasted for 24 h and dosed in the following manner: 500, 1000, 1500, 2000, 2500, 3000 mg/kg body weight. After the dosing, the rats were observed for 14 days for lethargy, jerkiness, and death [36].

### 2.8. DPPH Assay

To assess the antioxidant activity of the crude extract, photo-colorimetric methods were used to determine the free radical DPPH (2,2-Diphenyl-1-picrylhydrazyle) [37]. Samples of the ethanolic plant extract were diluted to 500, 1000, 1500, 2000, 2500, and 3000 ppm; 1 mL of each sample was added to the DPPH solution and prepared up to 5 mL with methanol, and then incubated for 40 min. Mixtures were kept at 25 °C, and the measurements were carried out using a spectrophotometer at 517 nm (spectrophotometer UV-340 G, Gehaka Santa Clara, CA, USA), and ascorbic acid was used as a standard. The tests were performed three times for all samples, and the mean value was calculated.

### 2.9. Biochemical Analyses

Blood samples were screened to determine various biochemical parameters.

### 2.10. Measurement of Plasma and Urine Sodium and Potassium Levels

For the screening of electrolytes or an acid–base imbalance, the electrolyte profile was used to check the effects of any treatment. Electrolytes include sodium, potassium, chloride, and bicarbonates for the diagnosis of any condition or disease [38]. Sodium and potassium concentrations in plasma and urine were measured by using a flame photometer (Sherwood Model 410, Nottingham, UK).

The samples were diluted (1:200) for the measurement of sodium in urine and plasma samples, and for the measurement of potassium in plasma samples, the same dilution was used, but for the potassium levels in urine, the dilution was 1:1000. All samples were measured in triplicate.

### 2.11. Urine Flow Rate

The urine flow rate is the amount of urine excreted per unit time and it was calculated using the following formula in µL/min/100 g of BW:

Urine output (mL/24 h) × 1000 × 100

Urine flow rate (µL/min/100 g of BW) = 1440 × Wt. of animal (grams)

### 2.12. Measurement of Plasma and Urine Creatinine Levels

Creatinine is a catabolic product of creatine phosphate in muscles, and depending upon the muscle mass, it is usually produced at a fairly constant rate in the body [39].

Plasma and urinary creatinine concentrations were measured spectrophotometrically (Jaffe’s reaction) by a method described before [40].

Urinary samples were diluted up to 50 times with distilled water. Both plasma and urine samples were deproteinized using trichloroacetic acid (1.2 M/L) along with centrifugation, and the supernatant was used for the measurement. The principle of this assay is based on the reaction between creatinine in the sample and picric acid in an alkaline medium to form a colored complex. This complex can be detected by a spectrophotometer at 520 nm wavelength. The complex formation should be measured in a short period after preparation to avoid interference. The preparation of the assay component is shown below in the table. The total volume of the sample, blank, and standard was transferred to a 96-well microtiter plate and incubated for 20 min at room temperature. Following the incubation period, the absorbance of the mixture was measured using a microplate reader (Synergy HT BioTek ^®^ Pittsburgh, PA, USA). All samples were analyzed in duplicate, and the concentration of creatinine in plasma and urine was calculated using the following formula:

Abs. of sample—Abs. of blank × Conc. of standard

Plasma conc. (mg/dL) = Abs. of standard − Abs. of blank

Abs. of sample—Abs. of blank × Conc. of standard × 50

Urine conc. (mg/dL) = Abs. of standard—Abs. of blank

Abs = Absorbance.

### 2.13. Histopathological Analysis

The histopathological analysis of the kidney was performed using Olympus light microscopy (×400) with an Olympus digital camera. Kidneys preserved in formalin were dehydrated, cleared in xylene, and placed in paraffin, and the kidney was cut into 5 µm sections and stained with periodic acid Schiff and hematoxylin. First, specific sections (5 µm each) of the kidneys were rehydrated and desalinized with alcohol. Next, these sections were treated with a particular amount of endogenous peroxidases for 0.5 h at 37 °C and were rinsed three times in phosphate buffer saline (PBS) at pH 7.00. After rinsing, the kidney sections were heated with 0.01 M sodium citrate buffer at pH 6.00 for 25 min and incubated for 1 h with 1% BSA. After incubation, the sections were condensed with hematoxylin, dehydrated with alcohol, and cleaned with xylene [37,38].

### 2.14. Statistical Analysis

One-way analysis of variance (ANOVA) and Bonferroni’s post hoc analysis were performed for all data (mean ± SEM (*n* = 5)) to determine the methodological significance between different experimental groups. Statistically, significance was considered at *p* < 0.05 [36].

## 3. Results

### 3.1. Phytochemical Analysis and Antioxidant Activity

The phytochemical examination of the ethanol plant extract showed that flavonoids, phenolic compounds, tannins, and saponins were present, whereas triterpenoids, steroids, and anthocyanins were not detercted. The results of different antioxidant assays showed that the concentration-dependent DPPH radical scavenging activity of the ethanol extract of *A. camelorum* was highest at 3000 mg/mL (96.07%), whereas the lowest (26.07%) was measured at 500 μg/mL. The highest percentage decrease was measured at 3000 μg/mL with respect to the power reduction of the plant extract (108.9 percent). At a lower concentration of 500 μg/mL, the lowest percentage reduction power (60.1 percent) was measured. The nitric oxide activity of *A. camelorum* showed that increasing concentrations of the extract markedly increased the nitric oxide scavenging. At 3000 μg/mL of the plant extract, the highest activity was observed. In hydrogen peroxide scavenging, when the plant extract concentration increased, the activity of scavenging increased. The highest activity at 3000 μg/mL and the lowest activity at 500 μg/mL were recorded. The SOD test results showed a higher percentage of inhibition of the plant extract at 3000 μg/mL (79.8%) and a lower percentage at the dose of 500 μg/mL (21.0%). Table 1 shows the phytoconstituents of the ethanol extract, while Table 2 summarizes the antioxidant activity of various assays.

### 3.2. HPLC Analysis

**Figure 1:**
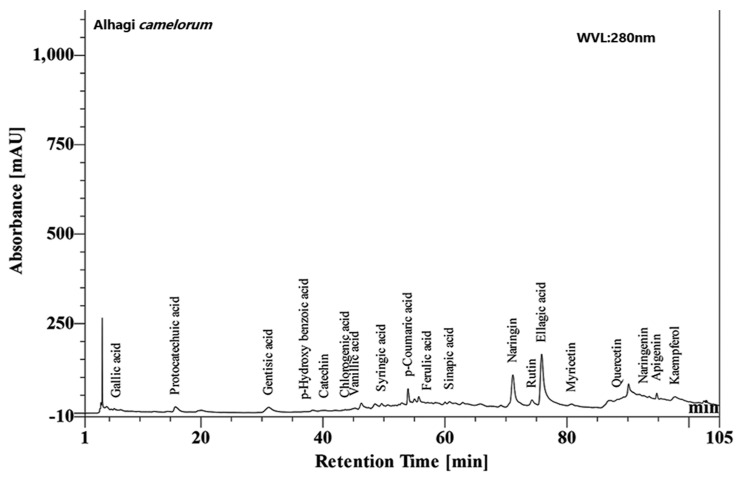
HPLC chromatogram of the hydroalcoholic plant extract of *Alhagi camelorum* showing gallic acid, rutin, etc.

The HPLC analysis revealed many phytoconstituents in varying concentrations at a 280 nm wavelength, as shown in (Figure 1).

### 3.3. Acute Oral Toxicity Dose Test

The current study was performed in compliance with OECD. Guideline 423 found that the maximum dose was preserved at 3000 mg/kg. The acute oral toxicity of the extract was evaluated in 24 rats. They were divided into six groups, and each group contained four rats that were fasted for 24 h and then dosed in the following manner: 500, 1000, 1500, 2000, 2500, and 3000 mg/kg body weight. After the dosing, the rats were observed for 14 days for lethargy, jerkiness, and death

#### Relative Organ Weight

Table 3 shows the average relative organ weights for rats treated with the maximal dose of the plant extract (6000 mg/kg). The relative weights of the kidneys, liver, heart, lungs, stomach, and spleen were not considerably different between the control and the treatment group.

### 3.4. Effect of the A. camelorum Plant Extract on the Body Weight and Plasma K of Cis-Treated Rats

Nephrotoxicity is caused by a unilateral dose of cisplatin (5 mg/kg i.p) that was biochemically shown by increases (*p* ≤ 0.05) in body weight, urine output, urinary sodium, urinary potassium, plasma creatinine, and kidney weight. In contrast, the cisplatin caused a decrease (*p* ≤ 0.05) in plasma Na and K and urinary creatinine. Table 4 shows the body and plasma potassium levels between the control and cisplatin treatment and after the experiment with Ac extracts on days 0, 7, 14, and 21 after dose administration. Compared with the control, there was a decrease in body weight after cisplatin treatment on days 7, 14, and 21. Treatment with Ac protected against a loss in body weight. It was observed that the body weight increased up to the 14th day and then started to decrease, and maximum B.W. and plasma potassium were measured after 14th days of observation with both plant extracts. It was also observed that higher dose administration of the extract increased the B.W. and plasma potassium levels of rats compared to a lower dose. However, the decrease in B.W. and plasma potassium indicated the presence of nephrotoxicity.

### 3.5. Effect of Ac Extracts on Plasma Creatinine and Plasma Sodium of Cis-Treated Rats

Table 5 presents the effect of Ac extracts on urinary creatinine and plasma sodium of cisplatin-treated rats. Results were observed after 0, 7, 15, and 21 days of dose administration. In addition, at 600 mg/kg, co-administration of Cis + AC affected the urinary creatinine and plasma sodium after the 21st day of observation. Overall, cisplatin treatment resulted in increased plasma creatinine and decreased plasma sodium; however, the co-administration of Cis + Ac^a^ and Cis + Ac^b^ significantly reduced the plasma creatinine and increased plasma sodium at rates of 400 and 600 mg/kg. However, it was concluded that Cis + Ac^a^ and Cis + Ac^b^ at 600 mg/kg showed better results than those observed at 400 mg/kg.

### 3.6. Effect of Ac on Urine Output and Urinary Na in Cis-Treated Rats

Table 6 shows the effect of the Ac plant extract on urine output and urinary sodium (Na) in Cis-treated rats after 0, 7, 14, and 21 days of dose administration. Overall, cisplatin-treated rats showed a reduction in urine output and urinary Na; however, co-administration of Cis + Ac^a^ and Cis + Ac^b^ significantly increased urine output and urinary Na at a dose of 400 and 600 mg/kg. However, it was concluded that Cis + Ac^a^ and Cis + Ac^b^ at the 600 mg/kg rate showed better results than those observed in response to 400 mg/kg.

### 3.7. Effect of Ac on Urinary K and Urine Flow Rate in Cis-Treated Rats

To further analyze the effect of the Ac extract, the level of urinary potassium (K) and urine flow rate were determined in each group of Cis-treated rats. Table 7 shows the results of the effect of the Ac extract on urinary K. The Cis-treated group showed a significant increase in urinary K from the first day to 21 days of observation compared to the control. Results revealed that the 400 and 600 mg/kg co-administration of Cis + Ac significantly reduced urinary K after 7 and 21 days of observation compared to the Cis group.

### 3.8. Effect of Ac on Urinary Creatinine and the Urinary Na/K Ratio in Cis-Treated Rats

Table 8 shows the nephroprotective effects of Ac on urinary creatinine and the urinary Na/K ratio in Cis-treated rats. The cis-treated rats showed a decline in urinary creatinine and an increase in the urinary Na/K ratio. It was observed that Cis + Ac co-administration at the rate of 600 mg/kg significantly increased urinary creatinine and decreased the urinary Na/K ratio level after 7, 14, and 21 days compared to cisplatin-treated rats; 600 mg/kg of Cis + Ac/significantly increased urinary creatinine and decreased the urinary Na/K ratio compared to the Cis group and the 400 mg/kg concentration.

### 3.9. Impact of Ac Extracts on Kidney Weight in Cis-Treated Rats

Table 9 shows the Ac extract nephroprotective results on kidney weight in every group. Compared to normal regulation, the kidney weight of animals regarded as Cis was very high. Co-administration of the extract and Cis at varying concentrations led to a reduction in kidney weight.

### 3.10. Histopathological Effects of Ac Extract in Cis-Treated Rats

**Figure 2:**
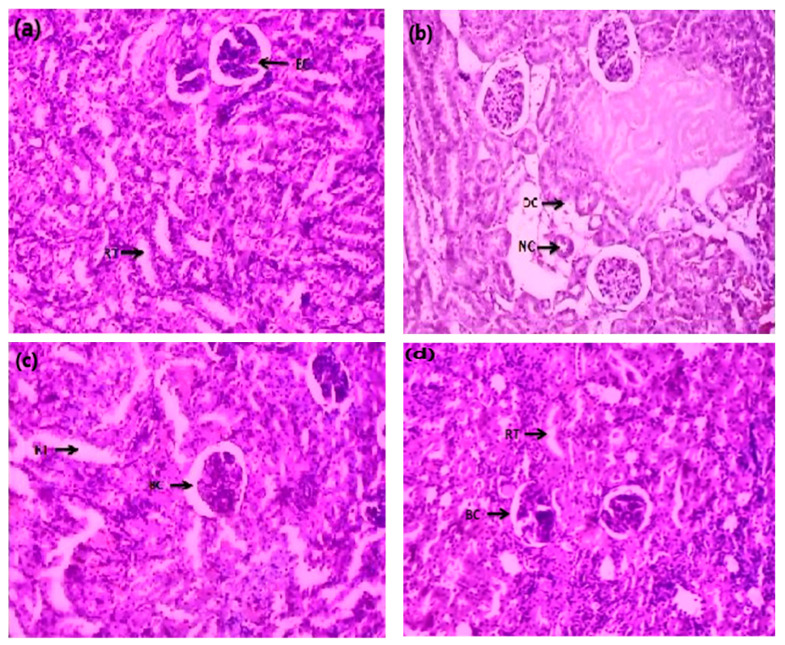
Photomicrograph of kidney sections of rats in the (**a**) control, (**b**) Cis, (**c**) Cis + AC (400 mg/kg), and (**d**) Cis + AC (600 mg/kg) groups. BC (Bowman’s capsule), RT (renal tubules), DC (distal convoluted tubule), NC (necrosis).

The histopathological effects of AC were examined by H&E staining for histopathological abnormalities. Results showed that the normal kidney showed uniform tubules and normal glomeruli covered with an epithelial layer and showed no blockage, bleeding or interfacial injury. In the Cis group, distorted histology with atrophied glomerulus and collecting tubules with necrosis were observed. Several degenerative changes were also observed in the Cis group in the form of atrophic lining with tubular and eosinophilic casts in cytoplasmic vacuolization of cells. In addition, glomerular hypertrophy was also noted in Cis-treated rats. However, Cis + AC co-administration at the 400 mg/kg rate showed normal glomeruli with mild histopathological results of damage in tubules. On the other hand, Cis + AC co-administration at 600 mg/kg rate significantly reduced the histopathological abnormalities induced by Cis. Minor renal damage was found in the proximal and distal tubules compared to Cis + AC-treated groups as shown in Figure 2.

## 4. Discussion

Cisplatin is a commonly used platinum-containing antineoplastic drug used to treat solid tumors, including those in the breast, lung, head, and neck [41,42]. Despite its multiple advantages in cancer treatment, its uses are limited due to nephrotoxicity [43,44]. With increasing use of Cis, drug-based nephrotoxicity has been increasing day by day and causes almost 26% of acute kidney injuries (AKI) [45]. The emerging evidence suggested that a single dose of Cis up to 50 mg/m^2^ induces side effects in the kidney; however, an estimation across 40% of patients that received Cis higher than this limit suffered from acute or mild renal dysfunction [46,47]. It was observed that Cis-induced nephrotoxicity led to renal vasculature that alters renal hemodynamics [48].

Cis is modified due to intracellular hydration to form a reactive metabolite and alters the expression of many water channels and membrane transporters to inhibit the function of mitochondria, ultimately blocking ATP production and leading to nitrosative and oxidative stress [49]. These pharmacological effects lead to the reabsorption and uncoupling of water that precedes the excretion of electrolytes, including magnesium (Mg), sodium (Na), calcium (Ca), potassium (K), and calcium (Ca). Moreover, Cis attacks different organelles and interfaces in DNA replication, altering several biological mechanisms, including necrosis, apoptosis, inflammation, and tubular derangement [40,47]. Mechanisms through which Cis causes nephrotoxicity are complex and involve different biological pathways such as oxidative stress, apoptosis, and inflammation [48]. ROS production is increased by Cis in mitochondria; NADPH oxidase and the cellular xanthine oxidase system are involved in the pathogenesis of Cis-induced severe kidney failure [17]. The function of various renal antioxidant enzymes, including catalase (CAT), glutathione (GHx), and superoxide dismutase (SOD), is also reduced by Cis [49]. However, based on the side effects of Cis, there is a need to develop a drug to reduce the pathophysiology of Cis. Nowadays, a mixture of different chemicals and natural products are used as potential Cis-neuroreceptors to interfere with the nephrotoxicity of Cis [50].

In the present study, the synergistic effects of Cis with AC plant extracts were screened at 400 and 600 mg/kg. They showed a significant effect against Cis-induced nephrotoxicity, but the mechanism of action is not fully understood and may involve reducing inflammation, oxidative stress, or apoptosis. The results of the present study revealed that Cis significantly reduced the bodyweight of rats by increasing the kidney weight. On the other hand, the co-administration of Cis + AC at 400 and 600 mg/kg significantly (*p* < 0.001) increased the body weight and reduced the kidney weight (*p* < 0.005). The weight loss in the Cis group was strongly related to insufficient nutrition, an increase in metabolic processes, metabolic imbalances, or mental conflict in the Cis-treatment community [51]. In addition, Cis induced tubular necrosis through increased kidney weight in groups treated with Cis due to ischemia or proliferation [49]. In animals treated with Cis + AC (600 mg/kg), there was a substantial reduction in kidney weight similar to a previous study [52]. Similar results were observed by Singh et al. [53], who revealed that co-administration of Cis (30 mg/kg) + morin hydrate (40 mg/kg) significantly reduced the Cis-treated rat kidney weight as compared to the Cis group. Similarly, Sahu et al. [54,55] reported that supplementation of Cis + bai at the 50 mg/kg rate significantly decreased the relative kidney weight and increased the body weight compared to the Cis group. They also observed a significant reduction in the plasma creatinine level to almost equal to that in the control.

The present study showed that co-administration of Cis + AC extract at 400 and 600 mg/kg significantly (*p* < 0.001) improved renal function (Table 1, Table 2, Table 3, Table 4, Table 5, Table 6, Table 7, Table 8 and Table 9); 400 and 600 mg/kg supplementation of Cis + Ac successfully (*p* < 0.005) increased the urinary sodium (Na), and potassium (K) level up to the control compared to the Cis-group that decreased the plasma Na and K levels after a single dose at 5 mg/kg Cis. In comparison, 400 and 600 mg/kg co-administration of Cis + AC improved (*p* < 0.005) the plasma creatinine, Na, and K levels that were reduced after intake of Cis. The weakening of membrane pumps such as Na-K is due to the nephrotoxicity caused by Cis. It leads to a decrease in salt reabsorption and hence increases the urine level [55,56,57]. In the present study, the Cis-group displayed hypernatriuria and hyperkaliuria. Co-administration of Cis + AC (400 and 600 mg/kg) allowed sodium and potassium levels to decrease to near normal values relative to the Cis groups (Table 6 and Table 7).

The results indicated that the AC extract has high nephroprotection. The best outcomes were noted after the 7th day of the experiment compared to the 14th and 21st days of observation. Our results are in agreement with the findings of Chtourou et al. [58]. They revealed that co-administration of Cis + Naringin100 significantly reduced the serum creatinine level up to 0.47 ± 0.02 mg/dL in rats compared to the Cis- group that showed a serum level of 0.97 ± 0.02 mg/dL after 5 mg/kg administration. Similarly, they observed that co-administration of Cis + Naringin (100 mg/kg) increased the urine creatinine level (6.27 ± 0.92 mg/dL) compared to the Cis group (4.05 ± 0.12 mg/dL). Fatima et al. [59] demonstrated that co-administration of Cis + A20 (EGCG + CoQ10) reduced the serum creatinine level (1.36 ± 0.30 mg/dL) compared to the Cis group (3.13 ± 0.25 mg/dL), with urine Na, K, Ca^2+^, and Mg^2+^ levels of 110 ± 2.56, 28 ± 3.01, 4.83 ± 0.05, and 27.4 ± 2.2 µmol/24 h, respectively.

Moreover, the histopathological analysis was performed to confirm the effect of both doses of AC on renal function. The results showed that after Cis (5 mg/kg) administration, the kidney exhibited glomerular hypertrophy, cytoplasmic vacuolization of cells, and atrophic lining with tubular and eosinophilic casts. However, after administration of Cis + AC at the rate of 400 and 600 mg/kg, a significant improvement was observed, indicating that Cis + AC effectively reduced the renal abnormalities associated with a single injection of Cis.

## 5. Conclusions

Based on our findings, it can be concluded that *Alhagi camelorum* seems to be safe and have high medicinal value. The ethanol extract showed the presence of flavonoids, phenols, tannins, and saponins and significantly high antioxidant activity. The treated rats did not show any anatomical, physiological or histopathological changes compared to the control. Kidney tissues appeared normal after the maximum dose of the extract with a possible alteration of distal tubules, proximal tubules, and glomerulus in the kidney tissue. The results of reversing Cis-induced nephrotoxicity suggest high potential for the extract for renal damage treatment, and most of the parameters retained to normal values after the administration of AC for only three weeks. The *A. camelorum* ethanol extract has great potential as an antioxidant and nephrotoxic therapeutic agent. Further studies are required to explore the exact molecular mechanism responsible for its nephroprotective effect, and LC-MS/MS spectrum analysis is recommended for further characterization.

## Figures and Tables

**Table 1:** Phytoconstituents in the ethanol extract of the *A. camelorum* plant.

Serial No	Test	Ethanolic Extract
1	Flavonoids	+++
2	Phenols	+++
3	Tannins	+++
4	Saponins	+++
5	Triterpenoids	-
6	Steroids	-
7	Anthocyanins	-

+++ = Highly present, - = absent.

**Table 2:** Outcomes of the antioxidant activity of the A. *camelorum* plant extract using the disparate test.

Concentration (μg/mL)	Inhibition of the Extract					Inhibition of the Standard				
	DPPH	Reducing Power	NO	H_2_O_2_	SOD	DPPH	Reducing Power	NO	H_2_O_2_	SOD
500	26.07	60.1	29	26	21.0	31.26	62.4	31.7	28	20.2
1000	46.60	71.6	39.4	36	31.68	51.47	71.7	43.3	40	33.7
1500	61.89	87.05	51.3	51	48.18	64.53	84.8	54.6	52.6	44.08
2000	79.64	91.3	62	68	59.3	83.49	91.6	66.8	70	64.10
2500	88.24	96.9	71.6	94	66.2	88.97	95.8	75.5	102	71.87
3000	96.07	108.9	75.9	128	79.86	96.93	105.8	79.8	131.6	86.03

DPPH (2,2-Diphenyl-1-picrylhydrazyl), NO (Nitric oxide), SOD (superoxide dismutase), H_2_O_2_ (Hydrogen peroxide).

**Table 3:** The impact of the *A. camelorum* plant extraction the weight of organs of the rats.

Treatment	Control Group	Treatment Group
Stomach	0.93 ± 0.10	0.92 ± 0.28
Heart	0.76 ± 0.13	0.72 ± 0.48
Liver	5.34 ± 0.76	6.26 ± 0.76
Kidney	3.4 ± 0.32	3.5 ± 0.35
Spleen	1.47 ± 0.35	1.65 ± 0.39
Lung	1.06 ± 0.49	1.93 ± 0.29

Organ/body weight (%). Mean ± SEM values (*n* = 5).

**Table 4:** Effect of crude extracts of Ac on B.W & plasma potassium of cisplatin-treated rats.

Body Weight (g)	Observation (Day)			
Groups	0	7	14	21
*A. camelorum* (Ac)				
Control	264 ± 9.7	286 ± 10.4	348 ± 10.2	326 ± 10.7
Cis	268 ± 9.3	206 ± 10.1 (*)	209 ± 11 (*)	184 ± 10.6 (*)
Cis + Ac^a^	287 ± 10.6	230 ± 9.3 (&)	267 ± 10.8 (&)	250 ± 12 (&)
Cis + Ac^b^	289 ± 10.8	240 ± 7.7 (#)	304 ± 10.6 (#)	289 ± 10.3 (#)
Plasma potassium (mEq/L)				
Control	8.3 ± 0.58	7.9 ± 0.69	8.5 ± 0.58	8.3 ± 0.48
Cis	8.0 ± 0.32	3.1 ± 0.44 (*)	4.6 ± 0.45 (*)	4.6± 0.52 (*)
Cis + Ac^a^	8.0 ± 0.66	5.5 ± 0.46 (&)	5.8 ±0.48 (&)	5.1 ± 0.35 (&)
Cis + Ac^b^	8.3 ± 0.47	6.7 ± 0.44 (#)	6.3± 0.53 (#)	7.2 ± 0.57 (#)

Mean ± SEM (*n* = 6), where Cis is cisplatin (5 mg/kg i.p), Cis + Ac^a^ is cisplatin + *Alhagi camelorum* extract (400 mg/kg/21 days), Cis + Ac^b^ is cisplatin + *Alhagi camelorum* extract (600 mg/kg/21 days. The results are considered significant (*) if *p* < 0.005. * *p* < 0.05 vs. normal control, & *p* < 0.05 vs. cisplatin, and # *p* < 0.05 vs. cisplatin + *Alhagi camelorum* on corresponding days.

**Table 5:** Effect of crude extracts of Ac. on urinary creatinine and plasma Na of cisplatin-treated rats.

Plasma Sodium (mEq/L)	Observation (Day)			
Groups	0	7	14	21
Ac				
Control	163 ± 8.4	201 ± 8.3	202 ± 6.7	163 ± 8.7
Cis	172 ± 7.3	108± 10.4 (*)	135 ± 8 (*)	127± 9.2 (*)
Cis + Ac^a^	168 ± 7.2	147 ± 8.3 (&)	154 ± 7.6 (&)	148 ± 9.6 (&)
Cis + Ac^b^	173 ± 6.6	174 ± 7.9 (#)	179 ± 7.0 (#)	168 ± 8.8 (#)
Plasma creatinine (mg/dL)				
Control	2.4 ± 0.47	2.4 ± 0.47	2.6 ± 0.41	2.6 ± 0.56
Cis	2.5 ± 0.40	6.7 ± 0.30 (*)	7.7 ± 0.47 (*)	6.0 ± 0.59 (*)
Cis + Ac^a^	2.6 ± 0.41	5.7 ± 0.28 (&)	6.4 ± 0.58 (&)	6.4 ± 0.52 (&)
Cis + Ac^b^	2.6 ± 0.56	4.9 + 0.28 (#)	5.2 ± 0.30 (#)	5.2 ± 0.45 (#)

Mean ± SEM (*n* = 6), where Cis is cisplatin (5 mg/kg i.p), Cis + Ac^a^ is cisplatin + *Alhagi camelorum* extract (400 mg/kg/21 days), Cis + Ac^b^ is cisplatin + *Alhagi camelorum* extract (600 mg/kg/21 days. The results are considered significant (*) if *p* < 0.005. * *p* < 0.05 vs. normal control, & *p* < 0.05 vs. cisplatin, and # *p* < 0.05 vs. cisplatin + *Alhagi camelorum* on corresponding days.

**Table 6:** Effect of crude extract of Ac on urine output and urinary Na of cisplatin-treated rats.

Urine Output (mL)	Observation (Day)			
Groups	0	7	14	21
Ac				
Control	9.6 ± 4.0	9.4 ± 4.0	9.9 ± 3.0	9.0 ± 2.6
Cis	9.4 ± 4.0	38± 4.6 (*)	35 ± 6.4 (*)	40 ± 2.9 (*)
Cis + Ac^a^	10.4 ± 3.1	30 ± 4.0 (&)	29 ± 10.7 (&)	35 ± 2.6 (&)
Cis +Ac^b^	9.6 ± 3.0	21 ± 3.8 (#)	25 ± 3.9 (#)	29 ± 1.3 (#)
Urinary sodium (mEq/L)				
Control	197 ± 6.4	195 ± 10.6	201 ± 9.7	196 ± 10.8
Cis	194 ± 6.9	320 ± 26 (*)	323 ± 26 (*)	372 ± 21 (*)
Cis + Ac^a^	197 ± 10.4	286 ± 10.6 (&)	292 ±10.0 (&)	310 ± 26 (&)
Cis + Ac^b^	188 ± 8.6	243 ± 10.7 (#)	260 ± 11.0 (#)	270 ± 10 (#)

Mean ± SEM (*n* = 6), where Cis is cisplatin (5 mg/kg i.p), Cis + Ac^a^ is cisplatin + *Alhagi camelorum* extract (400 mg/kg/21 days), Cis + Ac^b^ is cisplatin + *Alhagi camelorum* extract (600 mg/kg/21 days. The results are considered significant (*) if *p* < 0.005. * *p* < 0.05 vs. normal control, & *p* < 0.05 vs. cisplatin, and # *p* < 0.05 vs. cisplatin + *Alhagi camelorum* on corresponding days.

**Table 7:** Effect of crude extracts of Ac on urinary K and urine flow rate of cisplatin-treated rats.

Urinary Potassium (mEq/24 h)	Observation (Day)			
Groups	0	7	14	21
*Alhagi camelorum* (Ac)				
Control	6.3 ± 0.59	5.5 ± 0.49	5.8 ± 0.59	4.0 ± 0.43
Cis	6.8 ± 0.60	6.8 ± 0.43 (*)	6.9 ± 0.53 (*)	7.6 ± 0.58 (*)
Cis + Ac^a^	6.9 ± 0.40	6.6 ± 0.44 (&)	5.9 ± 0.48 (&)	4.0 ± 0.40 (&)
Cis +Ac^b^	6.8 ± 0.76	2.9 ± 0.44 (#)	4.7 ± 0.35 (#)	5.3 ± 0.57 (#)
Urine flow rate (µL/min/100 g of B.W.)				
Control	4.5 ± 0.78	4.3 ± 0.67	3.9 ± 0.52	3.4 ± 0.65
Cis	4.4 ± 0.65	26± 3.6 (*)	26 ± 3.4 (*)	28 ± 3.4 (*)
Cis + Ac^a^	4.5 ± 0.67	9.8 ± 0.85 (&)	9.0 ± 0.72 (&)	8.7 ± 0.78 (&)
Cis +Ac^b^	3.3 ± 0.76	7.6 ± 0.97 (#)	6.2 ± 0.66 (#)	6.9 ± 0.59 (#)

Mean ± SEM (*n* = 6), where Cis is cisplatin (5 mg/kg i.p), Cis + Ac^a^ is cisplatin + *A. camelorum* extract (400 mg/kg/21 days), Cis + Ac^b^ is cisplatin + *Alhagi camelorum* extract (600 mg/kg/21 days. The results are considered significant (*) if *p* < 0.005. * *p* < 0.05 vs. normal control, & *p* < 0.05 vs. cisplatin, and # *p* < 0.05 vs. cisplatin + *Alhagi camelorum* on corresponding days.

**Table 8:** Effect of crude extracts of Ac. on urinary creatinine and the urinary Na/K ratio of cisplatin-treated rats.

Urinary Creatinine(mg/dL)	Observation (Day)			
Groups	0	7	14	21
*Alhagi camelorum* (Ac)				
Control	6.8 ± 0.57	7.4 ± 0.43	8.8 ± 0.72	8.2 ± 0.72
Cis	6.8 ± 0.57	4.2 ± 0.47 (*)	4.3 ± 0.44 (*)	3.6 ± 0.44 (*)
Cis + Ac^a^	7.0 ± 0.59	5.2 ± 0.45 (&)	5.8 ±0.47 (&)	5.5 ± 0.46 (&)
Cis + Ac^b^	6.7 ± 0.46	6.3 ± 0.46 (#)	6.3 ± 0.56 (#)	7.0 ± 0.39 (#)
Urinary Na/K ratio				
Control	5.5 ± 0.70	4.8 ± 0.70	5.5 ± 0.69	6.1 ± 0.68
Cis	7.5 ± 0.60	38 ± 3.7 (*)	43 ± 0.97 (*)	44 ± 6 (*)
Cis + Ac^a^	8.4 ± 0.90	26 ± 3.8 (&)	10.7 ± 0.95 (&)	24 ± 3.6 (&)
Cis +Ac^b^	6.3 ± 0.66	8.0 ± 0.86 (#)	6.0 ± 0.67 (#)	10 ± 1.3 (#)

Mean ± SEM (*n* = 6), where Cis is cisplatin (5 mg/kg i.p), Cis + Ac^a^ is cisplatin + *Alhagi camelorum* extract (400 mg/kg/21 days), Cis + Ac^b^ is cisplatin + *Alhagi camelorum* extract (600 mg/kg/21 days. The results are considered significant (*) if *p* < 0.005. * *p* < 0.05 vs. normal control, & *p* < 0.05 vs. cisplatin, and # *p* < 0.05 vs. cisplatin + *Alhagi camelorum* on corresponding days.

**Table 9:** Effect of crude extracts of Ac on kidney weight in Cis-treated rats.

Groups	Kidney Weight (g)
Control	0.65 ± 0.058
Cis	2.7± 0.035 (*)
Cis + Ac^a^	2.6 ± 0.0068 (&)
Cis +Ac^b^	0.98 ± 0.0066 (#)

Statistical analysis was carried out using one-way analysis of variance (ANOVA) in all groups on respective days followed by Bonferroni’s posthoc test. The results are considered significant (*) if *p* < 0.005. * *p* < 0.05 vs. normal control, & *p* < 0.05 vs. cisplatin, and # *p* < 0.05 vs. cisplatin + *Alhagi camelorum*.

## Abbreviations

DPPH	2,2-Diphenyl-1-picrylhydrazyl
SOD	superoxide dismutase
NO	Nitric oxide
H_2_O_2_	Hydrogen peroxide
Ac	*Alhagi camelorum*
CAT	catalase
SOD	Superoxide dismutase
GHx	Glutathione
EGCG	epigallocatechin gallate
CoQ10	Coenzyme Q10
Bai	Baicalein
